# Eight years of follow-up after laminectomy of calcium pyrophosphate crystal deposition in the cervical yellow ligament of patient with Coffin–Lowry syndrome: A case report

**DOI:** 10.1097/MD.0000000000004468

**Published:** 2016-08-07

**Authors:** Tadao Morino, Tadanori Ogata, Hideki Horiuchi, Shintaro Yamaoka, Mitsumasa Fukuda, Hiromasa Miura

**Affiliations:** a Spine Center, Ehime University School of Medicine; b Department of Pediatrics, Ehime University School of Medicine; c Department of Orthopedic Surgery, Ehime University School of Medicine, Shitsukawa, Tohon City, Ehime, Japan.

**Keywords:** calcium pyrophosphate, cervical myelopathy, Coffin–Lowry syndrome, yellow ligament

## Abstract

**Background:**

We report 8 years of follow-up after decompression to treat cervical myelopathy in a patient with Coffin–Lowry syndrome (CLS). CLS is a rare X-linked semidominant syndrome associated with growth and psychomotor retardation, general hypotonia, and skeletal abnormalities. In this patient, the spinal cord was compressed by calcium pyrophosphate crystal deposition in the cervical yellow ligament (YL). To date, only 1 report has described clinical features after surgery for calcified cervical YL in CLS.

**Methods:**

A 15-year-old male with tetraplegia secondary to compression of the cervical spinal cord induced by a hypoplastic posterior arch of C1 and calcification of the YL from C2 to C7 was treated surgically with laminectomy from C1 to C7. The patient's history, clinical examination, imaging findings, and treatment are reported. The patient was incapable of speech because of mental retardation, so he could not describe his symptoms. Gait disturbance worsened over the 2 months before admission to our hospital. At admission, the patient could not move his extremities, and tendon reflexes of the upper and lower extremities were significantly increased. Computed tomography of the cervical spine showed YL calcification from C2 to C7. Magnetic resonance imaging showed consecutive compression of the cervical spinal cord. We diagnosed quadriplegia secondary to cervical cord damage and performed emergency surgery.

**Results:**

During C1–C7 laminectomy, YL calcification in C2–C7 was observed. The calcification was confirmed as calcium pyrophosphate by crystal analysis. Quadriplegia gradually resolved, and almost disappeared by 2 weeks after the operation. Cervical hyperlordosis was observed in radiographs starting from 1 month after the operation, but it has not progressed and is not associated with any symptoms.

**Conclusions:**

The efficacy of decompression continued, and no postoperative complications have occurred during at least 8 years of follow-up.

## 1 Introduction

Coffin–Lowry syndrome (CLS) is a rare X-linked semidominant syndrome associated with growth and psychomotor retardation, general hypotonia, and skeletal abnormalities.^[1,2]^ Musculoskeletal abnormalities in patients with CLS, such as short stature, large and soft fingers, hypothenar crease, finger tapering, and chest wall deformity, have been reported. In particular, spinal abnormalities including thoracolumbar kyphosis or kyphoscoliosis, vertebral notching, and disc space irregularities, are frequently observed.^[3]^

Although numerous reports and reviews on CLS have been published, to date, there is only 1 report describing surgical treatment for myelopathy induced by calcium pyrophosphate crystal deposition (CPPD) in the cervical yellow ligament (YL). Here we report 8 years of follow-up after decompression to treat cervical myelopathy in a CLS patient with compressed spinal cord by CPPD in the cervical YL.^[4]^ In this report, we discuss results from 8 years of follow-up after laminectomy to treat tetraplesia in a patient with CLS.

## 2 Patient consent

The patient's parents signed informed consent for publication of this case report and any associated images. This case report was approved by the ethical committee of Ehime University.

## 3 Case report

### 3.1 History

A 15-year-old man with spastic tetraplesia diagnosed with CLS at age 3 was referred to our hospital. One year before admission, the patient's parents noticed that he could not walk long distances and had a spastic gait. His gait disturbance became worse over time. One month before admission, the patient could not walk even several meters and had decreased upper extremity locomotor activity, such as an inability to elevate his arms. At the time of admission, he could not get up and could only make slight movements of the distal potions of his extremities.

### 3.2 Presentation

**Figure 1:**
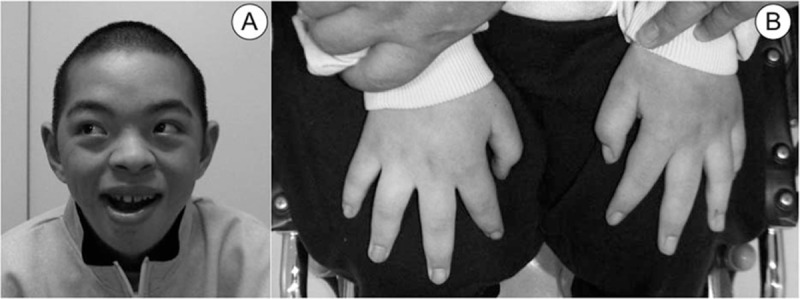
Appearance of the patient. The patient had characteristic findings, such as a prominent forehead, coarse faces, broad nose, thick septum, wide mouth, (A) and tapering fingers (B).

The patient's appearance was consistent with previous descriptions of patients with CLS. He had a prominent forehead, coarse faces, broad nose, thick septum, wide mouth, and tapering fingers (Fig. 1). He could emit a groan but could not have a conversation. We were unable to perform assess sensory function on physical examination. He could move his neck but not his trunk or extremities. Spastic weakness and very brisk muscle stretch reflexes were observed in all 4 extremities, and Babinski sign was observed. A balloon-catheter was placed because of dysuria. We suspected his symptoms were caused by cervical spinal cord damage, and performed imaging studies.

### 3.3 Examination

**Figure 2:**
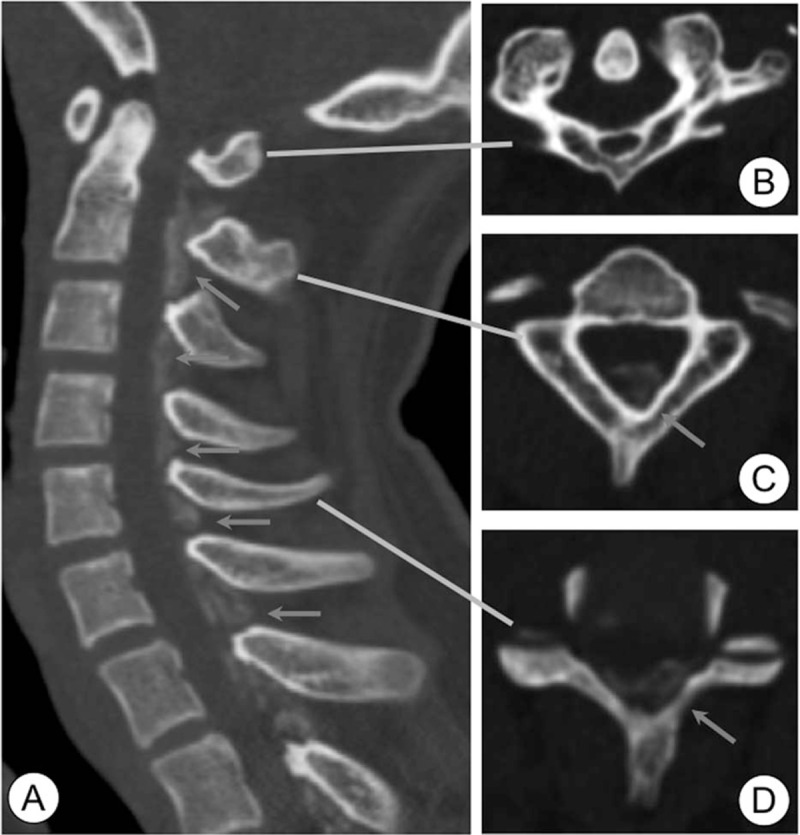
Preoperative computed tomography images. CPPD was observed in the yellow ligament from C1 to T2 in the sagittal image (A). Axial images of C1 (B), C2 (C), and C5 (D). Areas of CPPD are indicated by arrows. CPPD = calcium pyrophosphate crystal deposition.

**Figure 3:**
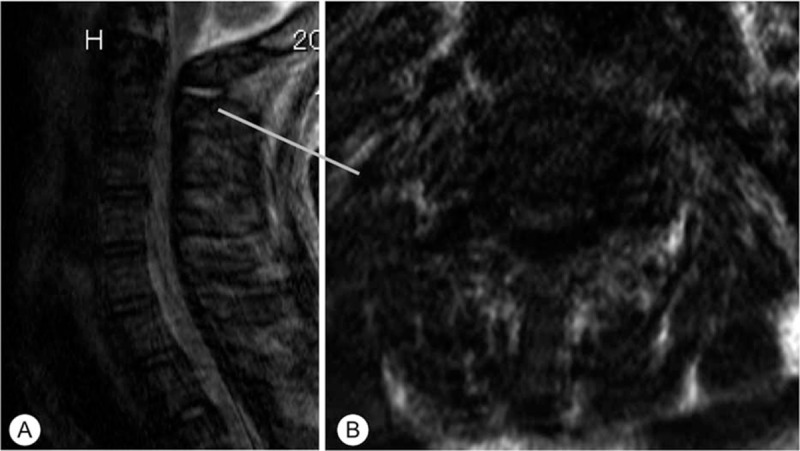
Preoperative magnetic resonance imaging. Sagittal T2-weighted images showing cervical spinal cord compression from C1 to C4 (A). T1-weighted imaging showed that the spinal cord was most compressed at the C1/2 level (B).

Computed tomography (CT) showed hypoplasia of the posterior arch of the C1 vertebra, and continuous calcification of the YL from C2 to T3 (Fig. 2). Magnetic resonance imaging (MRI) revealed that the cervical spinal cord from C1 to T1 was compressed by the calcification (Fig. 3). Image quality for MRI was low because the patient reacted poorly to anesthesia and moved during the examination. Although diagnostic information was limited, we diagnosed acute exacerbation of cervical myelopathy, and performed emergency surgery.

### 3.4 Surgery

**Figure 4:**
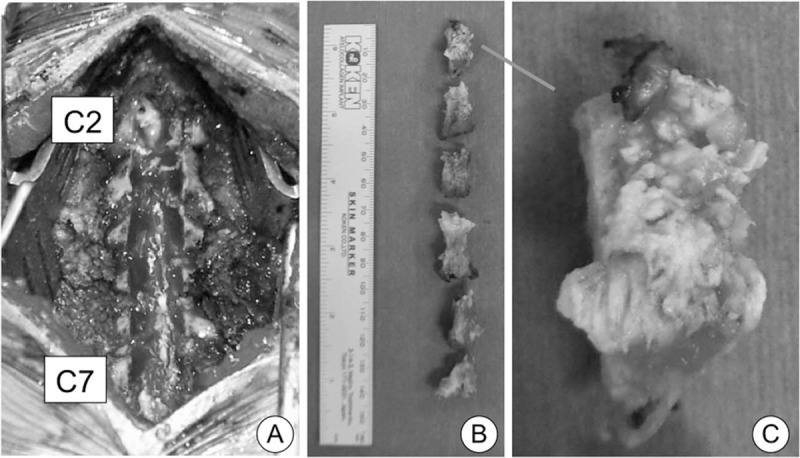
Perioperative images. Laminectomy was performed from C1 to C7 (A). View of the resected laminae from the ventral side, from C2 (top) to C7 (bottom) (B). Calcium pyrophosphate crystal deposition was observed in the yellow ligament on a magnified view of the C2 lamina (C).

Under general anesthesia in the prone position, the C1 to C7 laminae were exposed. Twenty millimeters of the width of the C2 to C7 laminae were removed using a high-speed drill. Adhesions between the calcification and dura mater were gently stripped off, and the laminae were resected en block with the calcification (Fig. 4). Fifteen millimeters of the width of the C1 posterior arch was removed using a high-speed drill. Pulsating dura mater was observed after laminectomy, but the pulse was weak. The dura mater appeared hypertrophic; however, we did not incise the dura mater.

### 3.5 Pathological findings

**Figure 5:**
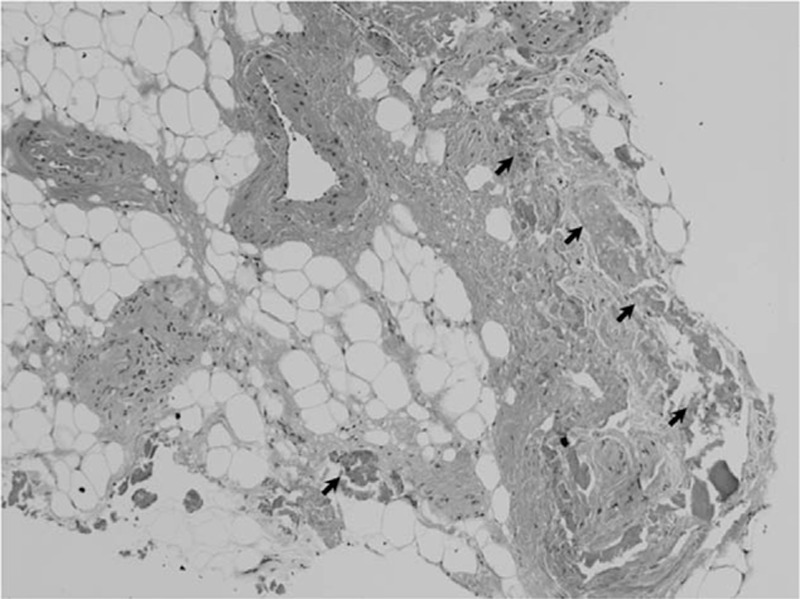
Pathological section (20 μm thickness) of the resected yellow ligament. Calcification is indicated by arrows. Hematoxylin and eosin staining (×10).

Calcification was observed in the YL (Fig. 5). Crystal analysis confirmed that the calcification consisted of calcium pyrophosphate and tricalcium phosphate.

### 3.6 Postoperative course

**Figure 6:**
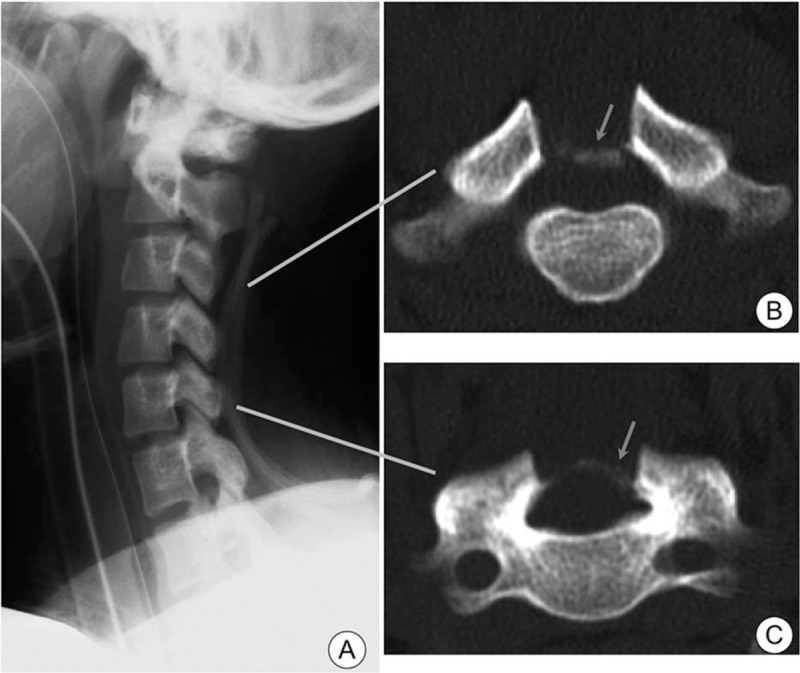
Postoperative images. C1 to C7 laminectomy was confirmed on the lateral radiograph (A). Calcification of the posterior part of the dura mater after laminectomy was observed on axial computed tomography images; representative slices at the level of C3 (B) and C5 (C) are shown.

A postoperative CT showed sufficient posterior decompression, although calcified dura mater was observed (Fig. 6). Movement of the extremities gradually improved after the operation. Two weeks after the operation, the patient could raise his arms over his head, have a meal with spoon, and walk with the assistance of a walker. After undergoing rehabilitation for 3 months, he could care for himself without any help in his daily activities. One year after the operation, he could run. Approximately 3 years after the operation, he fell down and developed increased spasticity of the lower limbs for 2 weeks, which resolved without any treatment. At that time, there was no worsening of symptoms in the upper limbs. We hypothesized that the temporary paralysis was caused by thoracic spinal cord damage, because there was canal stenosis secondary to ossification of the YL at the upper thoracic level. From this event to 8 years after the operation, his symptoms remained stable. Radiographs taken 1 week after the operation showed cervical hyperlordosis that has remained stable for 8 years. CT examination at 5 years after surgery showed progression of calcification around the dura mater but no spinal cord compression.

## 4 Discussion

Cases of CPPD in the cervical^[5,6]^ or thoracic^[7]^ YL have been reported. However, many studies were case reports. Mwaka et al^[8]^ reported 28 nonhereditary cases of CPPD in the cervical YL. The area affected by CPPD involved 1 to 4 intervertebral levels, and almost all of the patients had good clinical results after laminoplasty. The calcification could be classified as nodular or diffuse, and the longest continuous calcification was 4 vertebral levels long. Histological studies in the literature suggest the mechanism of CPPD in the degenerative YL involves chondrocyte hypertrophy and shrinkage of elastic fibers. Thus, CPPD commonly develops with aging and repeated mechanical stress in the YL.

In our patient with CLS, continuous CPPD developed from C2 to the upper thoracic level, at least 7 vertebral levels long. Differences in the area and type of calcification suggest that the etiology of calcification may be different in CLS. There has been no direct evidence that YL calcification in CLS is due to genetic factors.

We could find only 1 report describing cervical CPPD related to CLS that was treated with surgery.^[4]^ In that report, 2 patients with CLS underwent laminectomy to treat gait disturbance caused by cervical CPPD, and their gait improved.

**Figure 7:**
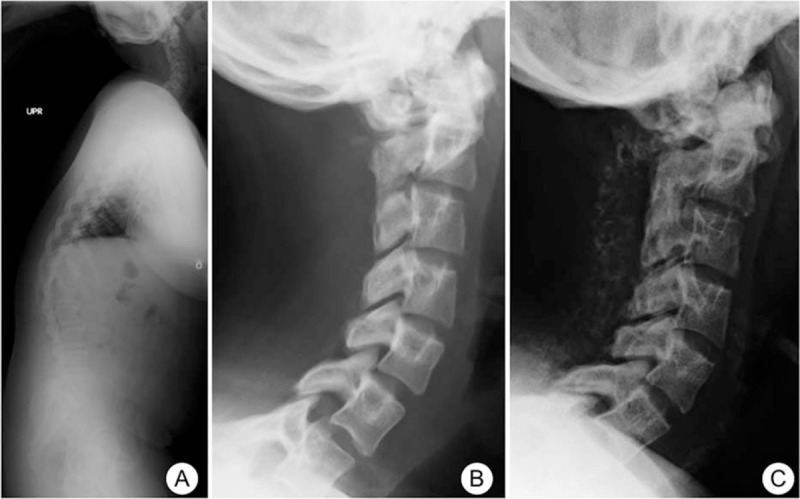
Lateral whole spine radiograph showing congenital thoracolumbar kyphosis and cervical lordosis (A). Cervical lordosis did not change from 1 y (B) to 8 y after surgery (C).

The authors did not explain why they selected laminectomy instead of laminoplasty. It is well known that cervical laminectomy results in a high rate of kyphosis deformity.^[9–11]^ However, we also selected laminectomy for this patient because there was a possibility of inadequate decompression and recurrent stenosis induced by progression of calcification, and recurrent stenosis is worse than a kyphosis deformity. Fortunately, kyphotic change did not develop during the 8-year follow-up period. However, lordosis occurred (Fig. 7). There was a kyphosis deformity in patient's thoracolumbar spine, and cervical hyperlordosis possibly developed in order to maintain sagittal balance.

Problems during the perioperative period include difficulties in performing preoperative examinations such as MRI or myelography in patients with CLS and intellectual disability, the possibility that patients with CLS have complications of cardiovascular disease such as mitral valve disease or dilated cardiomyopathy,^[12]^ and the possibility of patients pulling out a drainage tube or touching the surgical wound if they cannot understand instructions, which leads to a risk of infection. In fact, our patient received sedation for 3 days after the operation, and we could not evaluate neurological symptoms during that period.

We present a case of CLS with tetraplesia as a result of cervical myelopathy due to YL ossification. Laminectomy can be effective treatment, with at least 8 years of follow-up data.
